# The relation between sleep quality, sleep quantity, and gastrointestinal problems among colorectal cancer survivors: result from the PROFILES registry

**DOI:** 10.1007/s00520-021-06531-z

**Published:** 2021-09-14

**Authors:** Dounya Schoormans, Bonita van Es, Floortje Mols, Dareczka Wasowicz, Sandra Beijer, Nicole P. M. Ezendam

**Affiliations:** 1 Department of Clinical and Medical Psychology & Center of Research on Psychological disorders and Somatic diseases (CoRPS), Tilburg University, P.O.Box 90153, LE 5000 Tilburg, The Netherlands; 2 Department of Research and Development, Netherlands Comprehensive Cancer Organisation (IKNL), Utrecht, The Netherlands; 3 Department of Surgery, Elisabeth-TweeSteden Hospital, Tilburg, The Netherlands

**Keywords:** Colorectal cancer, Sleep, Gastrointestinal symptoms, PROFILES registry

## Abstract

**Purpose:**

Common residual symptoms among survivors of colorectal cancer (CRC) are sleep difficulties and gastrointestinal symptoms. Among patients with various gastrointestinal (inflammatory) diseases, sleep quality has been related to gastrointestinal symptoms. For CRC survivors, this relation is unclear; therefore, we examined the association between sleep quality and quantity with gastrointestinal symptoms among CRC survivors.

**Methods:**

CRC survivors registered in the Netherlands Cancer Registry—Southern Region diagnosed between 2000 and 2009 received a survey on sleep quality and quantity (Pittsburgh Sleep Quality Index) and gastrointestinal symptoms (European Organisation for Research and Treatment of Cancer, Quality of Life Questionnaire-Colorectal 38, EORTC QLQ-CR38) in 2014 (≥ 4 years after diagnosis). Secondary cross-sectional data analyses related sleep quality and quantity separately with gastrointestinal symptoms by means of logistic regression analyses.

**Results:**

In total, 1233 CRC survivors were included, of which 15% reported poor sleep quality. The least often reported gastrointestinal symptom was pain in the buttocks (15.1%) and most often reported was bloating (29.2%). CRC survivors with poor sleep quality were more likely to report gastrointestinal symptoms (*p*’s < 0.01). Survivors who slept < 6 h were more likely to report symptoms of bloating or flatulence, whereas survivors who slept 6–7 h reported more problems with indigestion.

**Conclusions:**

Worse sleep quality and short sleep duration were associated with higher occurrence of gastrointestinal symptoms.

**Implications for cancer survivors:**

Understanding the interplay between sleep quality and gastrointestinal symptoms and underlying mechanisms adds to better aftercare and perhaps reduction of residual gastrointestinal symptoms in CRC survivors by improving sleep quality.

## Introduction

Colorectal cancer (CRC) is worldwide one of the most common types of cancer [1]. Overall, incidence has been increasing and 5-year survival rates have been improving over the last two decades resulting in increasing numbers of CRC survivors [2]. CRC survivors are faced with the residual symptoms of both cancer and its treatment [3–5]. Two common residual symptoms among CRC survivors are sleep difficulties and gastrointestinal symptoms [3–5]. Both sleep difficulties and gastrointestinal symptoms have been associated with worse health outcomes (e.g., functioning and quality of life) in cancer survivors [3–5]. Sleep difficulties have been linked to heightened subjective perceptions of anxiety and pain among various cancer populations [5]. Moreover, gastrointestinal symptoms themselves can be a source of pain and physical discomfort, negatively affecting quality of life [3].

In patients with other gastrointestinal diseases such as inflammatory bowel disease, Crohn’s disease, and gastroesophageal reflux disease, sleep difficulties are related to gastrointestinal symptoms [6–8]. More specifically, poor sleep quality has been linked to exacerbations among patients with gastroesophageal reflux disease [6] and Crohn’s disease [7, 8]. Poor sleep quality involves poor subjective sleep quality, long sleep latency, short sleep duration, sleep inefficiency, sleep disturbances, use of sleep medication, and daytime dysfunction [9]. A short total sleep duration as a consequence of long sleep latency, waking after sleep onset or early waking, is common among cancer survivors [9, 10] and has shown to be associated with poor outcomes [11, 12].

Currently, it remains unknown whether there is an association between sleep quality/quantity and gastrointestinal problems in CRC survivors. The aim of this study was therefore to examine the association between sleep quality and quantity with gastrointestinal symptoms among CRC survivors. Gaining insight in the association between these common residual symptoms could optimize future treatment guidelines. Existing interventions for sleep problems may be more effectively applied, improving not only sleep quality and quantity, but also reducing the presence of gastrointestinal symptoms. In turn, reducing gastrointestinal symptoms may also improve sleep quality.

## Methods

### Participants

For this secondary data analyses, we used data from a longitudinal, population‐based cohort study including CRC survivors registered within the Netherlands Cancer Registry (NCR), which records data on all patients newly diagnosed with cancer [13]. Patients in the NCR—Southern Region, an area with 2.4 million inhabitants, were selected [14]. All patients diagnosed with stage I to IV CRC between 2000 and 2009 were eligible. Patients who had unverifiable addresses, had a cognitive impairment, died before the start of the study or were terminally ill, had stage 0 disease/carcinoma in situ, or were already included in another study (*n* = 169) minimizing patient burden and interference were excluded. A complete overview of the selection of patients can be found on our Web site under data and documentation (https://www.dataarchive.profilesregistry.nl/study_units/view/22). We used data from the fifth wave (questionnaires were sent between January 23, 2014, and May 21, 2014), as sleep quality and quantity were merely measured at this measurement occasion. Ethical approval for the study was obtained from the medical ethics committee of the Maxima Medical Centre in Veldhoven, the Netherlands (approval number 0822). All participants signed informed consent.

### Data collection

Data was collected via the Patient‐Reported Outcomes Following Initial Treatment and Long‐Term Evaluation of Survivorship (PROFILES) registry [15]. Survivors received a letter from their (former) attending specialist to inform them about the study. The letter included a link to a secure website, a login name, and a password so that interested patients could provide consent and complete questionnaires online. Those who preferred written communication could return a postcard, after which they received our paper and pencil informed consent form and questionnaire. Non-respondents were sent a reminder letter and a paper and pencil questionnaire within 2 months.

### Measures

#### Sociodemographic and clinical characteristics

Survivors’ sociodemographic (sex and age) and clinical information (tumor type, cancer stage, primary treatment, and time since diagnosis) were extracted from the NCR [14]. Information on marital status was self-reported. Comorbidity was assessed using yes/no statements to whether survivors experienced one or more of 14 chronic conditions using the Self-Administered Comorbidity Questionnaire [16].

#### Sleep quality and quantity

Sleep quality and quantity experienced in the *previous month* were measured using the 19-item self-reported Pittsburgh Sleep Quality Index (PSQI) [17]. The 19-items generate seven component scores: subjective sleep quality, sleep latency, sleep duration (i.e., quantity), habitual sleep efficiency, sleep disturbances, use of sleep medication, and daytime dysfunction. Each of these seven component scores is weighted equally on a scale ranging from 0 to 3, 0 indicating no difficulty and 3 indicating severe difficulty. Sleep quantity (i.e., sleep duration, “how many hours of actual sleep did you get at night?”) was reported and is scored into; > 7 h (0), 6–7 h (1), 5–6 h (2), and < 5 h (3) [17]. Overall, sleep quality (global PSQI score) was calculated by summing the seven component scores, which range from 0 to 21 [17]. Higher scores indicate worse sleep quality, and a global PSQI score > 8 is consistent with poor sleep quality among cancer populations [18].

#### Gastrointestinal symptoms

Gastrointestinal symptoms were assessed with the gastrointestinal tract symptom subscale of the European Organisation for the Research and Treatment of Cancer Quality of Life Questionnaire-Colorectal cancer 38 (EORTC QLQ-CR38) [19]. The 38-item EORTC QLQ-CR38 is applicable for patients who currently have or have survived CRC [19]. The subscale gastrointestinal tract symptoms consists of five items, which represent symptoms CRC survivors commonly suffer from having a bloated feeling in the abdomen, abdominal pain, pain in the buttocks, bothered by gas (flatulence), and belching. Each symptom was rated how often it appeared *last week*, ranging from 1 (not at all) to 4 (very much). For the current study, the gastrointestinal symptoms, “abdominal pain,” “pain in the buttocks,” “indigestions,” and “bloating,” were dichotomized into *not at all (answer category 1)* versus experiencing any symptoms (*answer categories 2, 3, or 4)*. As “flatulence” is a common symptom among healthy populations, we dichotomized it into *not at all* or *a little* (*answer categories 1 or 2)* versus *moderate* or *a lot* (*answer categories 3 or 4)*, to differentiate effectively among our sample of CRC survivors.

### Statistical analyses

Differences between respondents and non-respondents on all categorical sociodemographic (sex, age at time of questionnaire) and clinical characteristics (tumor type, cancer stage, primary cancer treatment, and time since diagnosis) were compared by means of chi-square tests. Descriptives for all sociodemographic (sex, age at questionnaire, and marital status) and clinical characteristics (tumor type, cancer stage, primary treatment, time since diagnosis, and comorbidities) were run. Logistic regression analyses were performed relating sleep quality and sleep quantity separately (independent variables) to each gastrointestinal symptom (i.e., bloating, abdominal pain, pain in buttocks, flatulence, and indigestion; dependent variables), while controlling for sociodemographic and clinical variables given their known associations with the (in)dependent variables. Logistic regression analyses including the original scoring of sleep quantity (> 7; 6–7; 5–6; < 5) were impossible due to the low number of CRC survivors in the category < 5 h of sleep (*n* = 32). Therefore, this category was merged into < 6 h of sleep. Analyses were performed using SPSS, version 24, and a significance level of 0.05 was used.

## Results

### Patient characteristics

**Fig. 1:**
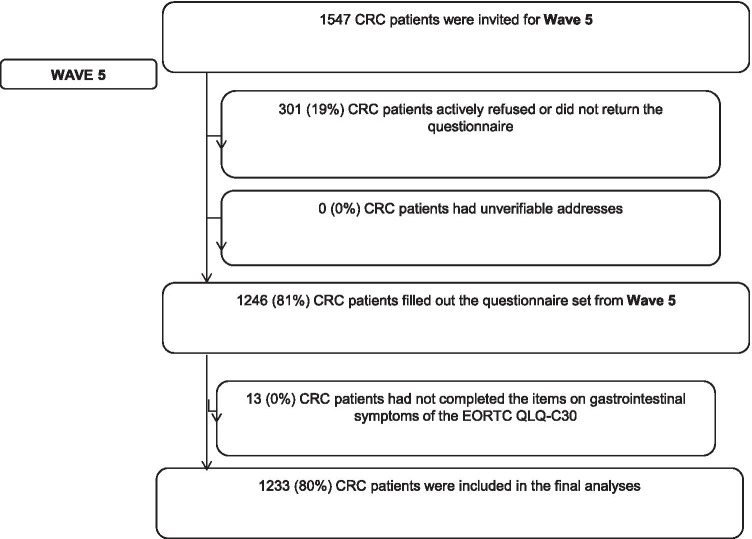
Flowchart of the study analysis

Of the 1547 eligible CRC survivors, 1233 (80%) responded and were included in our analyses (Fig. 1). No differences were seen between respondents and non-respondents on sociodemographic or clinical variables of interest (Table 1). Overall, 58.3% of CRC survivors were male (*n* = 719), the majority were older than 65 years (72.2%), and married or living together (76.6%). Nearly 60% were diagnosed with colon cancer (*n* = 727), and approximately one-third of survivors were diagnosed with a stage I CRC. Almost all survivors received surgery as a primary treatment (99.7%) (Table 1).

**Table 1:** Sociodemographics and clinical characteristics of respondents versus non-respondents

	Respondents (*n* = 1233)	Non-respondents (*n* = 301)	*P*-value
Male	719 (58.3)	183 (60.8)	0.43
Age			0.13
≤ 45	17 (1.4)	6 (2.0)	
46–50	19 (1.5)	10 (3.3)	
51–55	39 (3.2)	19 (6.3)	
56–60	90 (7.3)	20 (6.6)	
61–65	179 (14.5)	41 (13.6)	
66–70	261 (21.2)	60 (19.9)	
71–75	244 (19.8)	48 (15.9)	
76–80	233 (18.1)	53 (17.6)	
81–85	123 (10.0)	33 (11.0)	
≥ 86	38 (3.1)	11 (3.7)	
Marital status			–
Married/living together	932 (76.6)	–	
Single/divorced/widowed	285 (23.4)	–	
Tumor type			0.43
Colon	727 (59.0)	185 (61.5)	
Rectum	506 (41.0)	116 (38.5)	
Cancer stage			
I	390 (31.6)	76 (25.2)	0.22
II	427 (34.6)	119 (39.5)	
III	354 (28.7)	87 (28.9)	
IV	30 (2.4)	10 (3.3)	
Unknown	32 (2.6)	9 (3.0)	
Primary treatment			
Surgery	1227 (99.7)	298 (99.0)	0.12
Chemotherapy	369 (30.0)	99 (32.9)	0.33
Radiotherapy	400 (32.5)	98 (32.6)	0.98
Time since diagnosis			0.61
4–5 years	437 (35.4)	118 (39.2)	
6–7 years	227 (18.4)	54 (17.9)	
8–9	202 (16.4)	49 (16.3)	
≥ 10 years	367 (29.8)	80 (26.6)	
Number of comorbidities			–
0	336 (27.3)	–	
1	347 (28.1)	–	
2 or more	550 (44.6)	–	

Note: Numbers and percentages are presented

### Sleep quality, quantity, and gastrointestinal problems characteristics

**Fig. 2:**
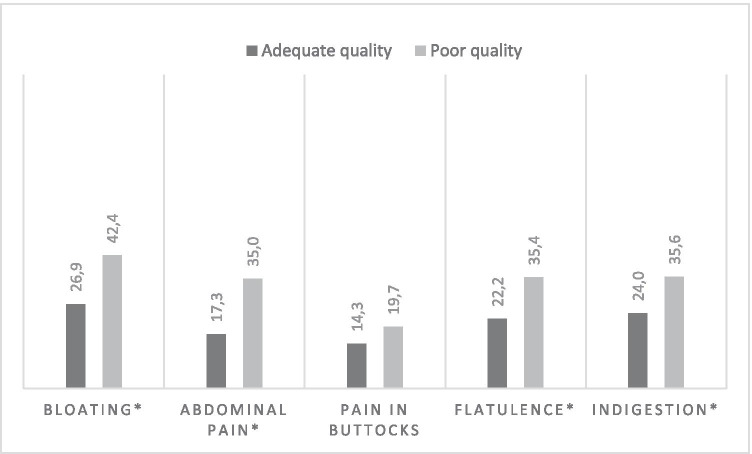
Percentage of CRC survivors who experience GI symptoms per sleep quality category (adequate versus poor). Note: Unadjusted comparisons *p-value < 0.01

**Fig. 3:**
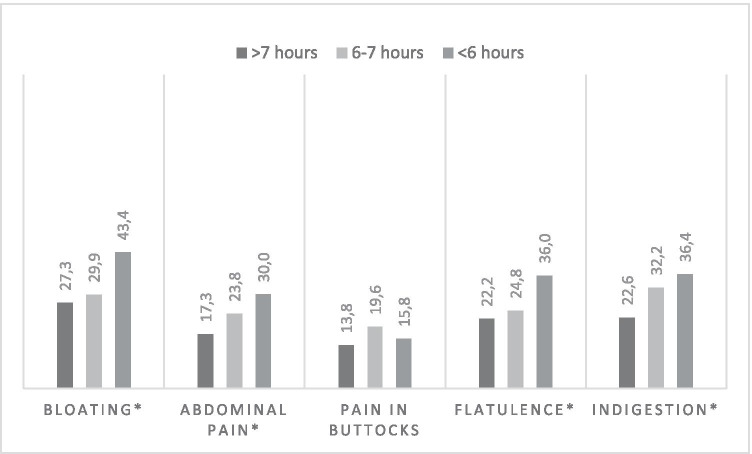
Percentage of CRC survivors who experience GI symptoms per sleep quantity (> 7 h, 6–7 h, < 6 h). Note: Unadjusted comparisons *p-value < 0.01

Fifteen percent (*n* = 181) of the CRC survivors reported poor global sleep quality. The majority reported > 7 h of sleep (73.1%, *n* = 869), whereas 18.3% (*n* = 217) reported 6–7 h, and 8.6% (*n* = 102) reported < 6 h of sleep. Regarding the gastrointestinal symptoms, 29.2% experienced bloating, 19.9% reported abdominal pain or pain in the buttocks (15.1%), nearly a quarter experienced flatulence (24.2%), and 25.7% reported symptoms of indigestion. CRC survivors who rated their sleep quality as poor or who reported a lower number of hours slept (quantity), reported more often gastrointestinal symptoms (i.e., bloating, abdominal pain, flatulence, and indigestion) (*p*’s < 0.01, Figs. 2 and 3).

### Associations between sleep quality, quantity, and gastrointestinal symptoms

CRC survivors who reported a poor global sleep quality were more likely to report symptoms of bloating (adjusted odds ratio (OR) = 1.13; 95% confidence interval (CI) = 1.08–1.18), abdominal pain (OR = 1.15; 95%CI = 1.09–1.20), pain in the buttocks (OR = 1.06; 95%CI = 1.01–1.12), flatulence (OR = 1.10; 95%CI = 1.06–1.15), and indigestion (OR = 1.11; 95%CI = 1.05–1.16) (Table 2). CRC survivors who reported sleeping 6–7 h a night were more likely to report symptoms of indigestion (OR = 1.50; 95%CI = 1.06–2.12) compared to survivors reporting > 7 h of sleep. CRC survivors reporting < 6 h of sleep were more likely to report symptoms of bloating (OR = 1.74; 95%CI = 1.11–2.73) and flatulence (OR = 1.84; 95%CI = 1.16–2.93) compared to CRC survivors who report > 7 h of sleep.

**Table 2:** Associations of sleep quality (total score and sleep quantity) with gastrointestinal symptoms by means of two logistic regression analyses

	Gastrointestinal symptoms				
	Bloating	Abdominal pain	Pain in buttocks	Flatulence	Indigestion
	OR (95%CI)	OR (95%CI)	OR (95%CI)	OR (95%CI)	OR (95%CI)
Poor sleep quality^§^	1.13 (1.08–1.18)*	1.15 (1.09–1.20)*	1.06 (1.01–1.12)*	1.10 (1.06–1.15)*	1.11 (1.06–1.16)*
Sleep quantity^¥^					
> 7 h (ref)	–	–	–	–	–
6–7 h	0.98 (0.70–1.39)	1.19 (0.80–1.75)	1.41 (0.93–2.15)	1.06 (0.73–1.52)	1.50 (1.06–2.12)*
< 6 h	1.74 (1.11–2.73)*	1.15 (0.90–2.51)	0.94 (0.50–1.77)	1.84 (1.16–2.93)*	1.56 (0.98–2.49)

Note: §Poor sleep quality < 8 versus adequate sleep quality ≥ 8. ¥Logistic regression analyses including the original scoring of sleep quantity (> 7; 6–7; 5–6; < 5) were impossible due to the low number of CRC survivors in the category < 5 h of sleep (*n* = 32). Therefore, this category was merged into < 6 h of sleep. Controlled for sociodemographic (sex, age at questionnaire, and marital status) and clinical characteristics (tumor type, cancer stage, primary treatment, time since diagnosis, and comorbidities)

## Discussion

Our results showed that CRC survivors reporting poor sleep quality were more likely to report gastrointestinal symptoms: bloating, abdominal pain, pain in the buttocks, flatulence, and indigestion. Furthermore, sleep quantity (i.e., hours of sleep) was related to experiencing gastrointestinal symptoms; CRC survivors who slept less than 6 h reported more symptoms of bloating or flatulence, whereas CRC survivors who slept 6–7 h reported more problems with indigestion. The findings of our study are in line with previous research [7, 20–23]. A cross-sectional study among the general population (*n* = 3228) showed that those with poor sleep quality were more likely to experience gastrointestinal symptoms [20]. Another study including 2674 people undergoing a health-check report that those with (35.8%) impaired sleep quality reported more gastrointestinal symptoms compared to those without impaired sleep quality [21]. Results from a population-based study (*n* = 2269) showed that the prevalence of irritable bowel disease was higher among those who reported experiencing sleep disturbances [22]. Among patients with inflammatory gastrointestinal diseases, similar associations have been reported [7, 23]. A longitudinal study among patients whose Crohn’s disease was in remission (*n* = 1291) showed that those with impaired sleep had twofold increased risk of active disease 6 months later [7]. The majority of studies are cross-sectional in nature and assume that impaired sleep quality precedes gastrointestinal symptoms; however, causality remains unclear. It can also be hypothesized that experienced gastrointestinal symptoms cause impairments in sleep quality.

Associations between sleep quality and gastrointestinal symptoms may be linked through immune functioning. We know that alterations of cytokines like interleukin-1 (IL-1), IL-6, and tumor necrosis factor-alpha (TNF-alpha) are known manifestations in various gastrointestinal diseases, including CRC [24]. Moreover, these cytokines are major contributors to sleep difficulties [25], and gastrointestinal symptoms [24]. In turn, sleep difficulties such as sleep deprivation are known to up-regulate these inflammatory cytokines [25]. Thus, poorer sleep may lead to elevated levels of pro-inflammatory cytokines, which in turn can contribute to the exacerbation of gastrointestinal symptoms.

This study has some limitations. The current sample consisted of CRC survivors 4 to 14 years after diagnosis; therefore, we introduced survivorship bias to our study. It has been indicated that poorer sleep quality is related to poorer overall survival; hence, those deceased CRC survivors may have had a poorer overall sleep quality [26]. Nevertheless, respondents versus non-respondents however showed no statistical differences on sociodemographic or clinical characteristics. Furthermore, we have no information on current disease status (e.g., recurrence or secondary cancers) nor whether survivors were on active treatment during questionnaire completion, while this could increase experienced gastrointestinal symptoms and sleep problems. Nonetheless, all included participants were diagnosed 4 years or longer ago, and the time since diagnosis was not related to experienced gastrointestinal symptoms nor sleep quality and quantity (data not shown). Furthermore, we examined time after diagnosis across tumor stages, to see whether stage IV CRC patients were more recently diagnosed and hence more likely to be receiving current treatment. Results showed that there was no relation between tumor stage and years after diagnosis indicating that our findings can be seen as robust. The cross-sectional design of these secondary data analyses prohibits us from making causal interferences. Although our analyses demonstrate a significant relation between sleep quality and quantity with gastrointestinal symptoms, the causality of the studied factors cannot be inferred. Hence, a bi-directional relationship between sleep quality/quantity and gastrointestinal problems remains plausible. CRC survivors may end up in a vicious circle of increased sleeping problems and gastrointestinal problems, especially as both are known to be driven by and resulting in increased inflammation [24, 25]. Nevertheless, knowledge on the possibly reciprocal relation between gastrointestinal symptoms and sleep quality is valuable for health-care providers and may result in awareness that besides asking about gastrointestinal symptoms, it is also important to ask for sleep problems in follow-up consultations. When patients mention one of both, it is important to explore the other component as both often co-occur. Furthermore, when trying to intervene on gastrointestinal symptoms, it may be valuable to also treat sleep problems or support patients to improve sleep quality and quantity as well, and vice versa.

Our study also poses several strengths, as this is the first study relating sleep quality and quantity to gastrointestinal symptoms among CRC survivors. Furthermore, our study has a high response rate (79.7%), has a large sample size (*n* = 1233), is representative of the total sample of CRC survivors given our population-based sampling method, and included sociodemographic and clinical characteristics as covariates.

## Conclusion

In conclusion, the present study showed that worse sleep quality and short sleep duration are associated with a higher occurrence of gastrointestinal symptoms (i.e., bloating, abdominal pain, pain in the buttocks, flatulence, and indigestion) among CRC survivors ≥ 4 years after initial treatment. Our results highlight the relative importance of improving sleep and gastrointestinal symptoms among CRC survivorship when it comes to healthy cancer survivorship. Ultimately, understanding the interplay between sleep quality/quantity and gastrointestinal symptoms and underlying mechanisms might allow for better aftercare.

## Data Availability

Data is freely available upon request for non-commercial use via www.profilesregistry.nl

## Code availability

Upon request
